# Draft genome sequence of a *Globisporangium polare* strain isolated from Antarctic moss

**DOI:** 10.1128/mra.01271-25

**Published:** 2026-05-28

**Authors:** Yuki Shima, Akira Ashida, Nanako Saga, Yoshiya Akimoto, Maurizio Camagna, Kevin K. Newsham, Masaki Uchida, Motoaki Tojo, Daigo Takemoto

**Affiliations:** 1 Graduate School of Bioagricultural Sciences, Nagoya University 12965, Nagoya, Japan; 2 Graduate School of Agriculture, Osaka Metropolitan University 12936, Sakai, Japan; 3 British Antarctic Survey (BAS), Natural Environment Research Council 41820 , Cambridge, United Kingdom; 4 National Institute of Polar Research (NIPR) 13554, Tachikawa, Tokyo, Japan; 5 Graduate Institute for Advanced Studies, SOKENDAI 13177, Tachikawa, Tokyo, Japan; University of Strathclyde, Glasgow, United Kingdom

**Keywords:** *Globisporangium polare*

## Abstract

*Globisporangium polare* is an oomycete species associated with mosses. Here, we report the draft genome sequence of *G. polare* strain 14-1, isolated from *Sanionia uncinata* (sickle-leaved hook-moss) on Adelaide Island, Antarctica.

## ANNOUNCEMENT

**Fig 1:**
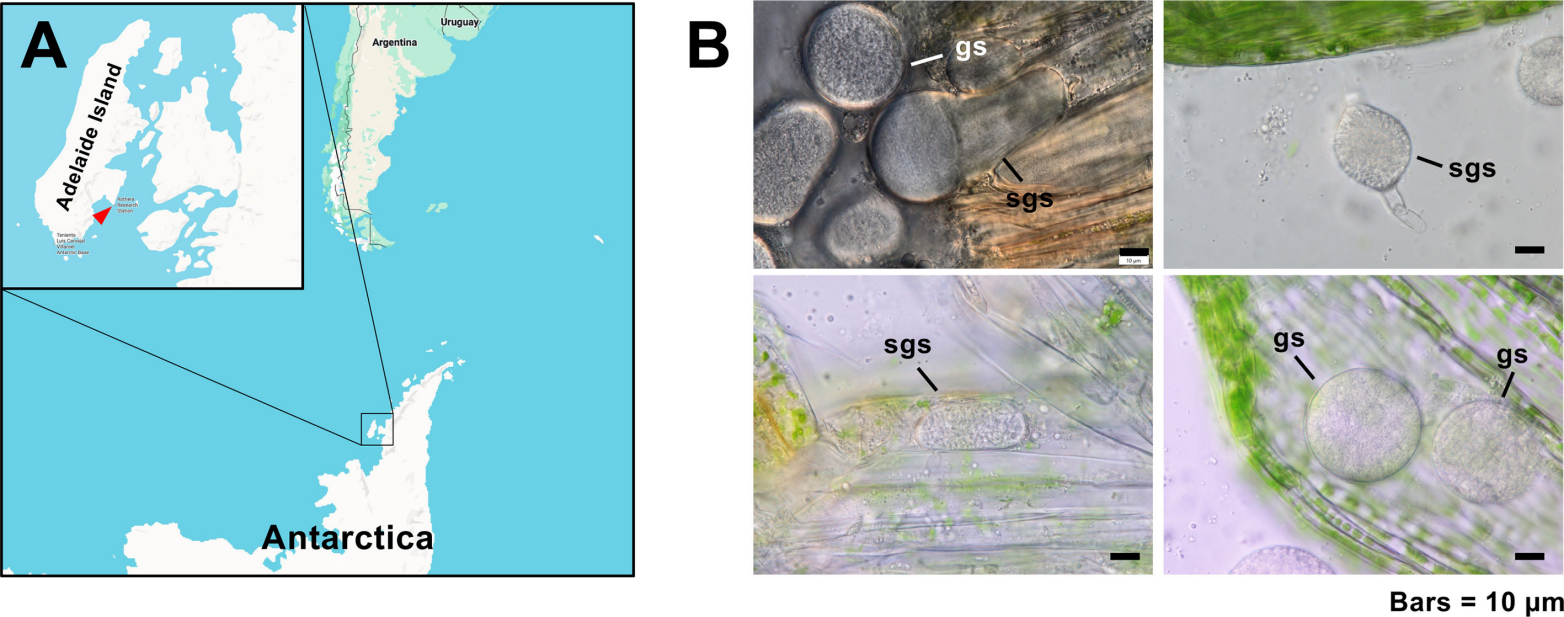
(**A**) Map of the Antarctic Peninsula showing the location from which *Globisporangium polare* strain 14-1, associated with *Sanionia uncinata*, was sampled (red arrowhead). The map was obtained from Google Maps. (**B**) Images of globose (gs) to subglobose (sgs) sporangia of *G. polare* developed in and around moss tissues.

*Globisporangium* is a genus of oomycetes belonging to the phylum Oomycota within the kingdom Straminipila. The genus was formerly classified within *Pythium*, but molecular phylogenetic analyses have demonstrated that it forms an independent monophyletic clade, leading to its reclassification as *Globisporangium* (1, 2). Species of this genus are widely distributed in soils and freshwater environments, and several species are known to infect a broad range of host plants (3, 4). *G. polare* is a psychrophilic oomycete isolated from mosses and freshwater in the Arctic and Antarctic, which has been reported to infect and persist in living plant tissues at low temperatures (5, 6). Here, we report the draft genome sequence of *G. polare* strain 14-1, which was isolated from the moss *Sanionia uncinata* (Hedw.) Loeske—a species adapted to cold and moist environments (7)—collected at Rothera Point, Adelaide Island, Antarctica (67°34′0″ S, 68°5′60″ W) in November 2007 (Fig. 1A). Shoots of *S. uncinata* were washed with water, placed on NARM medium (8), and incubated in darkness at 4°C. Emerging mycelia were grown on 1.5% water agar at 4°C, and each agar plug containing a single hyphal tip was transferred onto a 10% V8 juice agar plate at 4°C. The cultures were maintained in darkness at 2°C–10°C at Osaka Metropolitan University, Japan. Globose to subglobose sporangia were observed in and around moss tissues (Fig. 1B). For the genomic analyses, the strain was grown on potato dextrose agar at 23°C for 7 days and subcultured in potato dextrose broth at 23°C at 120 rpm for 4 days before fixation in 100% ethanol. DNA was extracted using a NucleoSpin Plant II kit (Takara Bio). Whole-genome sequencing libraries were prepared with a MGIEasy FS DNA Library Prep Set (MGI) and sequenced on a DNBSEQ-G400RS platform (2 × 150 bp). Unless otherwise noted, default parameters were used for all software. Reads were processed with fastp v0.24.0 (9) and assembled *de novo* using SPAdes v3.15.3 (10). Assembly quality was assessed with QUAST v5.0.2 (11). The summary of genome assemblies is shown in Table 1. Protein-coding genes were predicted with BRAKER3 v3.0.8 (12) using the oomycota_odb12 BUSCO data set for protein hints (13). Genome completeness was assessed with BUSCO v6.0.0 using oomycota_odb12 (13), yielding 99.1% complete BUSCOs. The ITS1 and mitochondrial *cox2* sequences were identified in the assembly by BLASTn using reference *Globisporangium* sequences were extracted as single contiguous sequences. The best BLASTn matches supported the assignment of strain 14-1 as *G. polare* (ITS1: 99.9% identity, 89.7% query coverage; *cox2*: 99.6% identity, 100% query coverage; best-hit accessions: ITS1, KJ716859; *cox2*, KJ595417 from *G. polare* CBS118203).

**TABLE 1:** Summary of *Globisporangium polare* genome assemblies and annotations

Strain	Host plant	No. of read pairs (spots)	N50	GC contents (%)	No. of total bases (bp)	No. of contigs	Sequencing coverage	No. of genes	Results from BUSCO analysis with data set oomycota_odb12			
									Complete BUSCOs (%)	No. of BUSCOs	No. of missing BUSCOs	No. of fragmented BUSCOs
14-1	*Sanionia uncinata*	11,527,521	44,986	57.9	39,404,733	3,348	174	15,063	99.1	3,431	11	21

## Data Availability

The draft genome assembly of *Globisporangium polare* strain 14-1 has been deposited in DDBJ/EMBL/GenBank under the whole-genome shotgun project BAAJAQ01 and is available as a genome assembly under accession number GCA_054786475.1 (assembly name: Gp_assembly_01). The raw sequencing reads are available in the DDBJ Sequence Read Archive under accession number DRR794741 and BioProject accession number PRJDB38039.

## References

[1] Thines M. 2018. Oomycetes. Curr Biol 28:R812–R813. doi:10.1016/j.cub.2018.05.062 30086308

[2] Uzuhashi S, Kakishima M, Tojo M. 2010. Phylogeny of the genus Pythium and description of new genera. Mycoscience 51:337–365. doi:10.1007/S10267-010-0046-7

[3] Liu Y, Vaghefi N, Ades PK, Idnurm A, Ahmed A, Taylor PWJ. 2023. Globisporangium and Pythium species associated with yield decline of Pyrethrum (Tanacetum cinerariifolium) in Australia. Plants (Basel) 12:1361. doi:10.3390/plants12061361 36987047 PMC10051369

[4] Molin C, Ribeiro NR, Matsumoto MN, Fernando Giasson N, Brollo J, Zanardo B, Pelissoni M, Capitanio S, Comín T, Deuner CC, et al.. 2021. Damping‐off of soybean in southern Brazil can be associated with different species of Globisporangium spp. and Pythium spp. Plant Pathol 70:1686–1694. doi:10.1111/ppa.13397

[5] Tojo M, van West P, Hoshino T, Kida K, Fujii H, Hakoda A, Kawaguchi Y, Mühlhauser HA, Van Den Berg AH, Küpper FC, et al.. 2012. Pythium polare, a new heterothallic oomycete causing brown discolouration of Sanionia uncinata in the Arctic and Antarctic. Fungal Biol 116:756–768. doi:10.1016/j.funbio.2012.04.005 22749162

[6] Tojo M, Fujii N, Yagi H, Yamashita Y, Tokura K, Kida K, Hakoda A, Herrero M-L, Hoshino T, Uchida M. 2021. Identification and isolation pattern of Globisporangium spp. from a Sanionia moss colony in Ny-Ålesund, Spitsbergen Is., Norway from 2006 to 2018. Microorganisms 9:1912. doi:10.3390/microorganisms9091912 34576807 PMC8467116

[7] Virtanen RJ, Lundberg PA, Moen J, Oksanen L. 1997. Topographic and altitudinal patterns in plant communities on European arctic islands. Polar Biol 17:95–113. doi:10.1007/s003000050111

[8] Morita Y, Tojo M. 2007. Modifications of PARP medium using fluazinam, miconazole, and nystatin for detection of Pythium spp. in soil. Plant Dis 91:1591–1599. doi:10.1094/PDIS-91-12-1591 30780596

[9] Chen S, Zhou Y, Chen Y, Gu J. 2018. Fastp: an ultra-fast all-in-one FASTQ preprocessor. Bioinformatics 34:i884–i890. doi:10.1093/bioinformatics/bty560 30423086 PMC6129281

[10] Bankevich A, Nurk S, Antipov D, Gurevich AA, Dvorkin M, Kulikov AS, Lesin VM, Nikolenko SI, Pham S, Prjibelski AD, et al.. 2012. SPAdes: a new genome assembly algorithm and its applications to single-cell sequencing. J Comput Biol 19:455–477. doi:10.1089/cmb.2012.0021 22506599 PMC3342519

[11] Gurevich A, Saveliev V, Vyahhi N, Tesler G. 2013. QUAST: quality assessment tool for genome assemblies. Bioinformatics 29:1072–1075. doi:10.1093/bioinformatics/btt086 23422339 PMC3624806

[12] Gabriel L, Brůna T, Hoff KJ, Ebel M, Lomsadze A, Borodovsky M, Stanke M. 2024. BRAKER3: fully automated genome annotation using RNA-seq and protein evidence with GeneMark-ETP, AUGUSTUS, and TSEBRA. Genome Res 34:769–777. doi:10.1101/gr.278090.123 38866550 PMC11216308

[13] Manni M, Berkeley MR, Seppey M, Simão FA, Zdobnov EM. 2021. BUSCO update: novel and streamlined workflows along with broader and deeper phylogenetic coverage for scoring of eukaryotic, prokaryotic, and viral genomes. Mol Biol Evol 38:4647–4654. doi:10.1093/molbev/msab199 34320186 PMC8476166

